# Low-Pass Filters for a Temperature Drift Correction Method for Electromagnetic Induction Systems

**DOI:** 10.3390/s23177322

**Published:** 2023-08-22

**Authors:** Martial Tazifor Tchantcho, Egon Zimmermann, Johan Alexander Huisman, Markus Dick, Achim Mester, Stefan van Waasen

**Affiliations:** 1 Central Institute of Engineering, Electronics and Analytics (ZEA-2), Forschungszentrum Juelich GmbH, 52428 Juelich, Germany; 2 Institute of Bio- and Geosciences Agrosphere (IBG-3), Forschungszentrum Juelich GmbH, 52428 Juelich, Germany; 3 Faculty of Engineering, Communication Systems, University of Duisburg-Essen, 47057 Duisburg, Germany

**Keywords:** electromagnetic induction (EMI), apparent electrical conductivity (ECa), temperature drift correction

## Abstract

Electromagnetic induction (EMI) systems are used for mapping the soil’s electrical conductivity in near-surface applications. EMI measurements are commonly affected by time-varying external environmental factors, with temperature fluctuations being a big contributing factor. This makes it challenging to obtain stable and reliable data from EMI measurements. To mitigate these temperature drift effects, it is customary to perform a temperature drift calibration of the instrument in a temperature-controlled environment. This involves recording the apparent electrical conductivity (ECa) values at specific temperatures to obtain a look-up table that can subsequently be used for static ECa drift correction. However, static drift correction does not account for the delayed thermal variations of the system components, which affects the accuracy of drift correction. Here, a drift correction approach is presented that accounts for delayed thermal variations of EMI system components using two low-pass filters (LPF). Scenarios with uniform and non-uniform temperature distributions in the measurement device are both considered. The approach is developed using a total of 15 measurements with a custom-made EMI device in a wide range of temperature conditions ranging from 10 °C to 50 °C. The EMI device is equipped with eight temperature sensors spread across the device that simultaneously measure the internal ambient temperature during measurements. To parameterize the proposed correction approach, a global optimization algorithm called Shuffled Complex Evolution (SCE-UA) was used for efficient estimation of the calibration parameters. Using the presented drift model to perform corrections for each individual measurement resulted in a root mean square error (RMSE) of <1 mSm^−1^ for all 15 measurements. This shows that the drift model can properly describe the drift of the measurement device. Performing a drift correction simultaneously for all datasets resulted in a RMSE <1.2 mSm^−1^, which is considerably lower than the RMSE values of up to 4.5 mSm^−1^ obtained when using only a single LPF to perform drift corrections. This shows that the presented drift correction method based on two LPFs is more appropriate and effective for mitigating temperature drift effects.

## 1. Introduction

Non-contact frequency domain electromagnetic induction (EMI) systems with small coil separations are widely used in geophysics to map the distribution of the electrical conductivity of the soil [1,2]. EMI instruments generally have at least one sender coil (Tx) and one or more receiver coils (Rx). The Tx generates a primary magnetic field ($H_{p}$) that penetrates the soil, causing eddy currents that generate a secondary magnetic field ($H_{s}$). At Rx the combination of $H_{p}$ and $H_{s}$ is measured, which contains information about the soil conductivity.

Many EMI instruments can perform measurements in both the horizontal co-planar mode (HCP) and the vertical co-planar mode (VCP). In HCP mode, the coils lie parallel to the soil with the magnetic dipole in a vertical direction. In VCP mode, the coils are orthogonal to the soil surface, and the magnetic dipole is parallel to the soil [3,4]. The HCP mode has approximately twice the exploration depth of the VCP mode. The sensitivity of the VCP mode is highest at the surface and decreases with depth, while the sensitivity of the HCP mode peaks at a depth of 0.4 times the intercoil separation [5].

A lot of progress has been made in the field of EMI system development and data interpretation, and many researchers have used the EMI technique in a broad range of applications. Some of the typical applications of EMI have been summarized by Allred et al. [6] and extensive reviews have been presented by Dennis Corwin [7] and by Corwin and Lesch [8]. In the field of agriculture, for example, Schmäck et al. [9] used EMI to analyze soil bulk density, volumetric soil water content, soil texture, and to predict zones of harmful soil compaction. Furthermore, Gebbers et al. [10] investigated the influences of seasonal variations and soil physico-chemical properties on soil electrical conductivity and related this to agricultural processes. EMI has also been used to investigate soil water content distribution. For instance, van’t Veen et al. [11] and Altdorff et al. [12] performed studies to relate EMI measurements to soil water content and water movement in the vadose zone. In other studies, EMI measurements were used to characterize soil clay content and soil textural heterogeneity [13].

Irrespective of the application for which EMI instruments are utilized, EMI data are known to be affected by systematic errors, which impact their accuracy. Such errors are very pronounced for EMI systems with coil separations smaller than 2 meters, where significant errors of several to dozens of mSm-1 have been observed when compared with reference data obtained from soil samples or more accurate contact-based conductivity measurements. This problem has been investigated in several studies with the aim of improving measurement accuracy. This problem has been investigated in several studies with the aim of providing solutions for improving measurement accuracy. For instance, Minsley et al. [14] and Sudduth et al. [15] attributed the deviations observed in EMI devices to incorrect instrument calibration and improper instrument leveling. Other known sources of systematic error are the presence of the operator [16] and cables lying close to the measurement system [10].

Furthermore, EMI devices are also known to be affected by temperature-dependent changes described commonly as drifts that vary unpredictably over time during measurements [17,18]. For instance, Huang et al. [19] tested the commercial DUALEM-41S and DUALEM-21S EMI instruments at varying temperatures and demonstrated that they affected the electrical conductivity values. Hanssens et al. [20] used ambient temperature variations to characterize the drift patterns of different electromagnetic instruments during static ground measurements. Mester et al. [21] identified several factors that thermally affect the properties of the system hardware, such as the thermal drift of coils resulting from daily ambient temperature variations. By exposing the commercial EM38, CM-138, and OhmMapper instruments to varying temperatures at a fixed position during measurements, Gebbers et al. [10] also reported drift effects associated with variations in air temperature. Sudduth et al. [15] investigated the effect of varying ambient temperatures from 23 °C to 35 °C on the accuracy of the commercial EM38 measurements and concluded that the effects are difficult to correct due to the non-linear relation between the drifts and ambient temperature.

The general consensus from these studies was that it is necessary to find measures to mitigate temperature drifts in EMI data. Several suggestions have been made to reduce the effects of temperature drifts. For instance, Huang et al. [19] suggested that the instrument should be shaded with non-conductive thermal insulation. Robinson et al. [17] attributed the drift effects observed to the differential heating of EMI devices, which results in a non-uniform temperature distribution, making it problematic to correct the resulting drifts. Abdu et al. [22] suggested that the EMI measurements should preferably be done on a cloudy day with less temperature variation. Tan X. [23] proposed the inclusion of a cooling system such as a fan during EMI measurements to minimize temperature drift.

In further attempts to mitigate temperature drift effects, Tan X. [23] identified two types of temperature drifts: drifts due to slow, uniform temperature variations and drifts resulting from fast temperature variations. The drifts resulting from slow ambient temperature changes are easier to correct. This is because when the temperature is changing slowly, the inner thermal changes of the instrument components (coil and electronics) also follow the measured ambient temperature, such that the delay due to the heating or cooling of the components can be corrected with the measured temperature [23]. The drifts due to quick ambient temperature changes, on the other hand, usually result in differences between the measured ambient temperature and the temperatures of different system components, which can be problematic to correct since components react with a delayed response to fast temperature changes. As a solution for drifts due to fast but uniform temperature variations, Tan X. [23] proposed to measure the temperature-dependent electrical properties of the coils to compensate for drifts due to slow reactions of the properties to fast temperature changes.

To eliminate the effects of drifts due to fast, uniform temperature variations, Tazifor et al. [24] proposed a dynamic approach that uses information from temperature sensors and a thermal drift model based on a low-pass filter to model and correct delayed drift effects of hardware components. They concluded that such a dynamic thermal characterization of the drift effects improved the overall accuracy compared with pure static characterization, which is solely based on using a simple look-up table for drift correction. The proposed approach was, however, only effective for EMI measurements performed under uniform ambient temperature variations.

In this paper, a model-based approach is proposed that can correct for drift effects resulting from fast, non-uniform temperature variations. It is designed for rigid-boom EMI systems with the Tx and Rx in one enclosure but can also be used for a modular EMI system under development, where the Tx and Rx are in different enclosures. In the following sections, the EMI measurement system consisting of a transmitter, three receivers, and integrated temperature sensors is introduced first. Next, the proposed drift model for drift correction based on low-pass filters is presented. This is followed by a description of the optimization method used to estimate the parameters of the drift model. Finally, the results of drift correction with the two low-pass filter models are presented and discussed. Comparisons are made with the previous dynamic model composing one low-pass filter, and at the end, conclusions are drawn.

## 2. Materials and Methods

### 2.1. Measurement System

**Figure 1:**
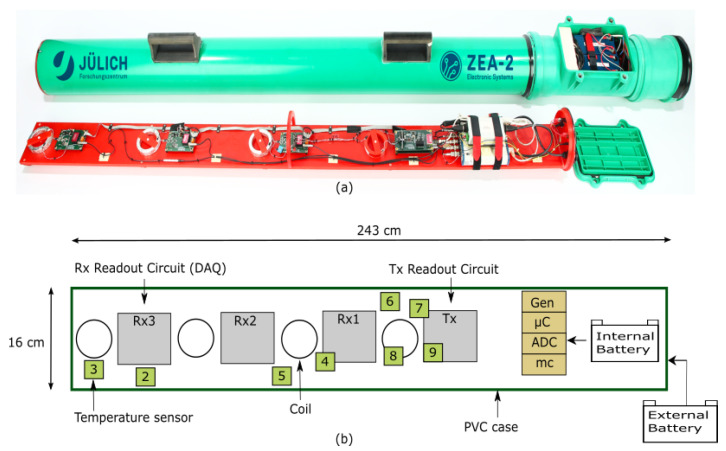
(**a**) Photo of the modified electromagnetic induction (EMI) instrument; (**b**) Representation of the measurement system consisting of a generator unit (Gen), a transmitter coil (Tx) and 3 receiver coils (Rx_1_, Rx_2_ and Rx_3_). The data acquisition unit (DAQ) consists of an analog to digital converter (ADC), a microcontroller (μC), a mini-computer (mc) and eight temperature sensors spread across the device. All components are enclosed in a polyvinyl chloride (PVC) casing. Temperature sensors 2 and 6 measure the PVC temperature, sensors 3, 4 and 5 measure the air temperature, sensor 7 measures the heat sink temperature, sensor 8 measures the Tx coil temperature and sensor 9 measures the printed circuit board (PCB) temperature of the Tx. The system has a length of 243 cm and a width of 16 cm and is powered by a 12 V battery.

The measurement system used in this study is shown in Figure 1. It is based on the system developed by Mester et al. [25] and described by Tan X. [23] as well as Tazifor et al. [24]. The system was developed for studying modular and scalable system concepts and for investigating interference effects, e.g., system drifts. It consists of a transmitter coil (Tx) and three receiver coils (Rx_1_, Rx_2_, and Rx_3_) separated by a distance (x) of 0.4 m between the respective coils. The analysis in this paper is based on Rx_3_ located at 1.2 m from the Tx. The generator (Gen) supplied by a 12 V battery powers the Tx with AC current at a frequency of 10 kHz. The voltage signal from the Rx_3_ coil and the current signal from the Tx coil are sent to the data acquisition unit (DAQ), consisting of a 24-bit analog-to-digital converter (ADC), a micro-controller (μC) used to configure the hardware, a mini computer (mc) containing the MATLAB-based measurement software, and eight temperature sensors spread across the measurement system. The entire setup is controlled by an external personal computer using a wireless local area network (WLAN).

A generator in the EMI measurement instrument provides a time-varying current through the Tx, which generates a primary magnetic field ($H_{p}$) that penetrates the soil. Based on the induction law, electrical voltages are induced in the soil, which produces so-called eddy currents. The magnitude of the currents depends on the electrical conductivity of the soil. The eddy currents, in turn, generate a secondary magnetic field ($H_{s}$) and the superposition of the $H_{p}$ and the $H_{s}$ is measured at the Rx. The ratio between the $H_{p}$ and the $H_{s}$ has a real and an imaginary component, whereby the imaginary component is related to the electrical conductivity of the sensed subsurface. The cumulative response of a certain volume of the underlying subsurface can be obtained as the average weighted electrical conductivity values over the sensed subsurface. This is typically converted into ECa using a Maxwell-based full solution electromagnetic forward model [26], the low induction number (LIN) approximation [5], or a combination of both [25,27].

The measurement system was used to perform 21 calibration measurements at different locations in the Research Center Jülich, Germany. The measurements were recorded in the summer periods of 2021 and 2022 and showed varying temperature ranges and variations. The temperature sensors in the device measured temperatures varying from about 10 °C to 50 °C during the calibration measurements. For each measurement, the device was raised 0.7 m above the ground using wooden supports, and data was acquired in the VCP configuration to further minimize soil effects. Tazifor et al. [24] demonstrated that for a measurement at a height of 0.7 m and an inter-coil spacing of 1.2 m, the expected ECa change due to soil temperature changes is about 0.07 mSm ^−1^ K^−1^ (worst-case). This is low compared with expected system drifts larger than 1 mSm^−1^K. For effective temperature drift analysis, only temperature data with a range of at least 10 K were considered (15 out of 21 datasets). Furthermore, only measured data after a warm-up time of 2 h were considered.

### 2.2. Drift Correction Model

**Figure 2:**
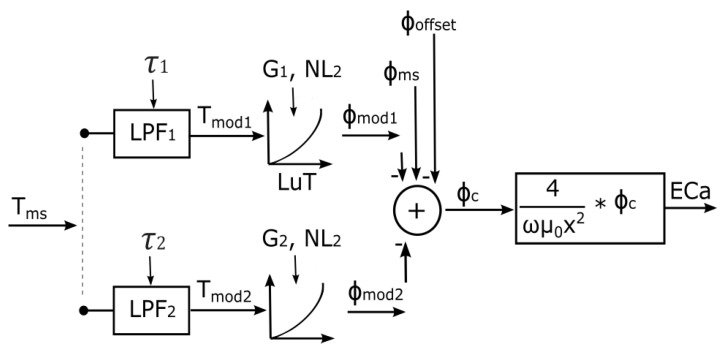
Phase drift model with pre-selected measured temperatures $T_{ms}$, which serve as input for the low-pass filters (LPF). The outputs of $LPF_{1}$ and $LPF_{2}$ are the modelled temperatures $T_{mod1}$ and $T_{mod2}$, which are converted to modelled phases $\Phi_{mod1}$ and $\Phi_{mod2}$ respectively by cubic spline interpolation using a lookup table (LuT).

The phase drift model shown in Figure 2 is used to model the temperature-dependent dynamic characteristics of the measurement system using two low-pass filters (LPF). The LPFs are used to estimate the delayed response of the internal temperature of the system components to external temperature variations. To facilitate the conversion of temperature information into phase values, a look-up table (LuT) with cubic spline interpolation is used. The combination of the two LPFs and the LuT constitutes the complete phase drift model, which is described in more detail subsequently. The calibration parameters that control the phase drift model are the time constant (τ) from the LPF, the gain (*G*), and the non-linear variable (NL) of the LuT, as well as the system phase offset ($\Phi_{offset}$).

The offset ($\Phi_{offset}$) is not determined in this work; rather, it can be determined after drift correction based on a method proposed by Tan et al. [28]. Their method simultaneously determines calibration parameters, including multiplicative and additive factors for different coil configurations, as well as an inverted 1D horizontally layered subsurface model consisting of electrical conductivity values and the corresponding thicknesses for each layer. Other methods for offset calibration have also been implemented by von Hebel et al. [27] who used electrical resistivity tomography with Dipole-Dipole and Schlumberger electrode arrays and vertical electrical soundings. All three methods obtained robust calibration results.

The drift model is based on the infinite impulse response (IIR) filter function described in detail by Tazifor et al. [24]. The inputs for the drift model are pre-selected measured temperatures $T_{ms}$, which are transformed into a delayed response $T_{mod}$ using the time constant parameter τ and the $T_{mod}$ of the previous time step:

(1) $$T_{mod}\left(t\right)=b_{0}\cdot T_{ms}\left(t\right)+b_{1}\cdot T_{ms}\left(t-1\right)+a_{1}\cdot T_{mod}\left(t-1\right)$$

where *t* are the discrete time points of the time series, $a_{1}$, $b_{0}$ and $b_{1}$ are the filter coefficients determined from τ and the sampling period $T_{s}$ using

(2) $$a_{1}=\frac{1-\frac{T_{s}}{2\cdot \tau }}{1+\frac{T_{s}}{2\cdot \tau }}\;and$$

(3) $$b_{0}=b_{1}=\frac{\frac{T_{s}}{2\cdot \tau }}{1+\frac{T_{s}}{2\cdot \tau }}.$$

The temperature range for the LuT was from 0 °C to 50 °C, based on the measured temperature range. To build the LuT, three reference temperature points are set: ($T_{ref_{min}}$ = 0.0 °C, $T_{ref_{mid}}$ = 25.0 °C, and $T_{ref_{max}}$ = 50.0 °C). The parameters *G* and NL are used to determine the corresponding reference phase values $\Phi_{ref_{min}}$, $\Phi_{ref_{mid}}$, and $\Phi_{ref_{max}}$. The initial calibration point is the phase measured at 0 °C, and it is set for convenience at zero since the phase offset shift has no effect on drift correction:

(4) $$\Phi_{ref_{min}}=0.$$

$\Phi_{ref_{max}}$ is obtained from the temperature range and the gain parameter *G*:

(5) $$\Phi_{ref_{max}}=G\cdot \left(T_{ref_{max}}-T_{ref_{min}}\right).$$

The determination of $\Phi_{ref_{mid}}$ also involves the non-linear term NL:

(6) $$\Phi_{ref_{mid}}=NL\cdot G\cdot \left(T_{ref_{mid}}-T_{ref_{min}}\right).$$

A value of 1 for the NL parameter implies a linear relationship between phase and temperature. An NL value different from 1 will result in a non-linear temperature-phase relationship. By using LuT and cubic spline interpolation, the modelled temperatures $T_{mod1}$ and $T_{mod2}$ for the two LPFs are converted into the modeled phases $\Phi_{mod1}$ and $\Phi_{mod2}$ respectively, as already described by Tazifor et al. [24]. The corrected phase $\Phi_{c}$ was then calculated from $\Phi_{mod1}$ and $\Phi_{mod2}$ as

(7) $$\Phi_{c}=\Phi_{ms}-\Phi_{mod1}-\Phi_{mod2}$$

The corrected phase can be converted to ECa using the approximation proposed by [5]:

(8) $$ECa=\frac{4}{\omega \mu_{0}x^{2}}\cdot tan\left(\Phi_{c}\right)$$

where *x* is the inter-coil spacing, ω is the angular frequency and $\mu_{0}$ is the permeability of free space. This equation is valid for low induction numbers.

### 2.3. Selection of Temperature Sensors

It is challenging to determine the most useful position to place the temperature sensors on the EMI device, as sensors on different components will react with different delays to external temperature changes. It is unclear whether the sensors should be placed on components with large thermal capacities (coils) that react slowly to temperature changes or simply in the air that reacts fast. For the EMI data analyzed here, there are two positions where drifts may originate. These are the positions of the Tx coil and the Rx coil at an inter-coil spacing of 1.2 m. In the Rx region, the air and PVC temperatures are measured using sensors 3 and 2, respectively. In the Tx region, the PVC, heat-sink, Tx coil, and PCB temperatures are measured using sensors 6, 7, 8, and 9, respectively.

In an attempt to find suitable sensors for drift correction, delayed responses of all eight measured temperature time series were modeled with the first part of the drift correction model shown in Figure 2 (i.e., excluding the LuT and only considering the LPFs). The goal was to check if temperatures with fast reaction times can be used to model temperatures with a delayed response, which would imply that both types of temperature time series could be used for correction. In addition, this analysis was used to check if the LPFs could properly model the system component delays. To ease analysis and facilitate the comparison between modeled and measured temperatures, the root mean square error was calculated after fitting the optimal value for the time constant (τ).

### 2.4. Assessment of Spatial Temperature Variation

To evaluate whether temperature drift correction with two LPFs allows for fast, non-uniform temperature variations, it is of interest to determine the temperature distribution within the measurement device. Here, we again consider the two regions where drifts are expected to originate. If the two regions have similar temperature variations, it is anticipated that a model with only one LPF will suffice to correct the drift, as shown in ref. [24]. If the temperature time series in the two regions differ, it is anticipated that a model with two LPFs will be required to correct the drifts. To evaluate the effect of an uneven temperature distribution on drift, the available calibration datasets were analyzed and separated into two classes: datasets with a uniform temperature variation (UTV) and datasets with a non-uniform temperature variation (NUTV). To separate the UTV and NUTV datasets, a principal component analysis (PCA) of the measured temperature data was used. The first step of the PCA method [29] consists of determining the covariance matrix

(9) $$C=T_{ms}^{T}\cdot T_{ms}$$

of the normalised (by $\sqrt{n}$) and mean-centered measured temperature time series $T_{ms}$, where *n* is the number of temperature time series [30]. An eigen decomposition of this covariance matrix

(10) $$\left[E_{vec},E_{val}\right]=eigen\left(C\right)$$

transforms the temperature data into eigenvalues $E_{val}$ with their corresponding linear independent (orthogonal) eigenvectors $E_{vec}$[31]. The eigenvalues are an indication of the magnitude of the respective eigenvectors and a measure of their importance in explaining variation within the dataset [30]. After calculation of the eigenvalues, they were normalized with the sum of all eigenvalues. These normalized eigenvalues ($E_{val,N}$) facilitate the comparison of different temperature time series with respect to their homogeneity. If the first normalised eigenvalue $E_{val,1N}$ is close to 1, all temperature time series show similar variation, which thus indicates a uniform temperature distribution. Here, a threshold value $V_{th}$ was used to differentiate between UTV and NUTV datasets. All temperature datasets with $E_{val,1N}$ greater than or equal to $V_{th}$ were classified as UTV datasets, and all datasets with $E_{val,1N}$ less than $V_{th}$ were classified as NUTV datasets.

### 2.5. Determination of the Representative Calibration Parameters

To estimate the calibration parameters m = ($\tau_{1}$, $G_{1}$, $NL_{1}$, $\tau_{2}$, $G_{2}$, $NL_{2}$) for the two LPFs, the misfit between the measured phase $\Phi_{ms}$ and the modelled phase $\Phi_{mod}$ was calculated using the objective function

(11) $$RMSE=\sqrt{\left|\right|\Phi_{c}-mean\left(\Phi_{c}\right){\left|\right|}_{2}}$$

based on the L2-norm. Here, the objective function RMSE is used for optimization without the offset (mean value). It should be noted that the drift model is not limited to only 2 LPFs but can be adapted to 3 or more as per requirement. In this case, three more parameters are added for every additional LPF.

Initial tests with local search algorithms showed that the optimization results were affected by local minima in the objective function, as indicated by different results for different starting values of the calibration parameters. For this reason, a global optimization method named shuffled complex evolution (SCE-UA) [32] was used to minimize the objective function. The SCE-UA algorithm is a stochastic optimization method that is commonly used to solve complex problems in a variety of fields, such as hydrology, environmental science, and engineering. The algorithm begins by creating an initial population of randomly sampled parameter sets from the feasible parameter space, which is the set of all possible solutions that meet the problem’s constraints. Based on the suggestion from Duan and Gupta [32], the initial population m×n is divided into *n* complexes, where *n* equals the number of calibration parameters and each complex contains a fixed number of parameter sets *m*. The parameter sets in each complex are evolved based on an extension of the simplex method [33].

After this, the parameter sets in each complex are again combined into a single population, which completes the first loop of the algorithm. In the next loop, the entire population is reassigned to different complexes to promote information sharing and prevent the algorithm from getting stuck in local minima. As the search progresses, the points in the population tend to converge towards the neighborhood of the global optimum, which is the best solution in the entire feasible space [32]. In this paper, the search was stopped when the objective function value did not improve by more than 1% in the last 20 loops.

A suitable set of calibration parameters should be able to correct all datasets. It is assumed that the intrinsic drift parameters are stable over longer periods of months or years and do not vary with time. If this were the case, it would be highly challenging to calibrate the system for drift. Therefore, all datasets were simultaneously fitted. Preliminary analysis showed that the models with more than one LPF showed strong dependencies between individual parameters. For example, it is possible to obtain the same overall *G* for several combinations of $G_{1}$ and $G_{2}$ when using two LPFs. It is therefore required to set adequate boundaries for the parameter space. To obtain such boundaries, the range of $\tau_{1}$, $G_{1}$, $\tau_{2}$, $G_{2}$ was determined for a linear version of the drift model by removing the NL term (i.e., setting $NL_{1}$ and $NL_{2}$ to 1). Wide boundaries were used for the remaining parameters: 0≤τ1,τ2≤4000 s, $-e^{-4}\leq G1,G2\leq e^{-4}$ radK^−1^.

To only consider data with approximately linear behavior, the calibration for the initial ranges considered only a subset of the data. In particular, only data were considered in a reduced temperature range around the mean temperature with a range of 10 K. Furthermore, only NUTV datasets was used to reduce the degree of dependence between the parameters because it is anticipated that the NUTV datasets need two LPFs for drift modeling. The range of the respective calibration parameters across all NUTV datasets were used to estimate new and smaller boundaries for the feasible parameter space. In the final step, the new boundaries were used to calibrate all datasets using the non-linear drift model and the full temperature range (0 °C–50 °C).

In the following, three types of calibrations were performed (named A, B, and C) to evaluate the performance of drift models with one and two LPF. In type A calibrations, all datasets were individually fitted with the objective function RMSE in Equation (11), using temperature measurements from sensors 3 and 9 and two LPFs. Type A calibrations are expected to provide the lowest fitting error and will serve as a reference. In type B calibration, all datasets were simultaneously fitted with the same temperature sensors and two LPFs. Finally, type C calibration only considered one LPF and the mean of temperature sensors 3 and 9 to perform simultaneous fitting on all datasets using the initial wide boundaries for the parameter space.

## 3. Results and Discussion

### 3.1. Selection of Temperature Sensors

**Figure 3:**
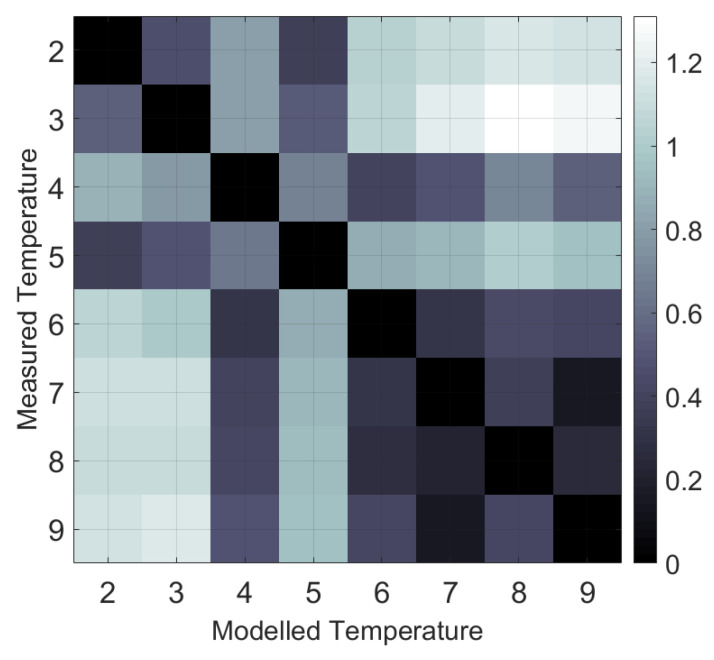
Root mean square error (RMSE) between modelled and measured temperatures to identify representative temperature sensors suitable for drift correction. The colour bar shows the RMSE between modelled and measured temperatures (in Kelvin). An error value of 0 indicates that one sensor can perfectly replace another temperature sensor.

The eight temperature sensors were fitted with each other using the LPF part of the drift model (i.e., only the time constant τ parameter was evaluated) to identify the most relevant temperature sensors suitable for drift correction (Figure 3). During the fitting run, the RMSE (Equation (11)) and the delay (τ) between the respective sensors were evaluated. It can be seen that the temperature sensors 2 and 3 in the Rx region result in a small RMSE (less than 0.5 K), whereby, sensor 3 models sensor 2 with a delay τ of 336 s. Furthermore, it can be seen that sensors 6, 7, 8, and 9 in the Tx region result in a small RMSE (less than 0.45 K), whereby sensor 9 models sensor 8 with a delay τ of 337 s. Sensor 7 was placed on the heat-sink and also showed a small error (less than 0.35 K), but it was not considered because the self-heating may not always be representative of the temperature in this region. Temperature sensor 4 in the middle region is more similar to the sensors in the Tx region, whereas temperature sensor 5 is more similar to the Rx_3_ region. However, there are no drift-relevant components in this middle region that have an influence on the drift behavior for the intercoil spacing of 1.2 m.

The results show that slow-reacting sensors placed on system components with large heat capacities, such as the Tx coil (measured by temperature sensor 8), can be modeled sufficiently well by sensors with a fast response. On the other hand, it is difficult to model sensors with a fast response using sensors with a slow response. Based on this analysis, temperature sensors 3 and 9 were selected as being representative for the Tx and Rx_3_ regions, respectively. These sensors are the fastest sensors, can properly model other sensors, and can therefore be used to replace them.

### 3.2. Assessment of Spatial Temperature Variation

**Figure 4:**
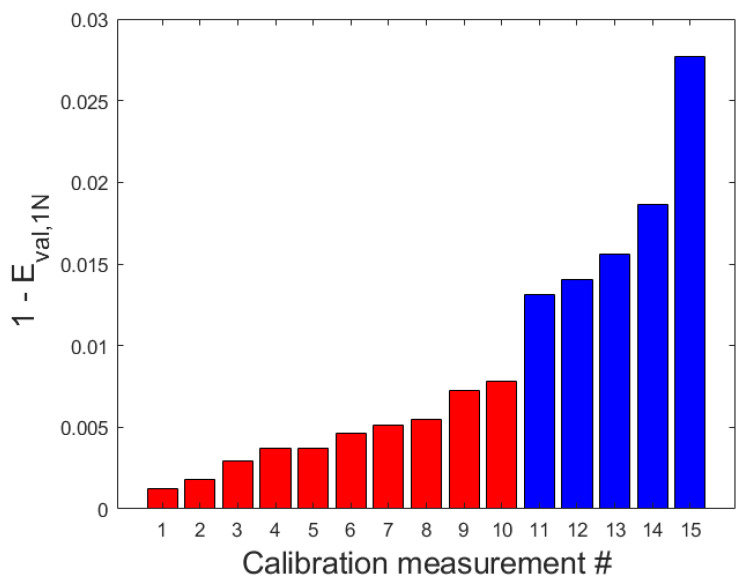
Plot of the residual eigenvalues (1-$E_{val,1N}$) for all datasets obtained from principal component analysis (PCA) on time series of temperature sensors 3 and 9. The red bars represent datasets recorded with uniform temperature distributions and the blue plots represent datasets recorded with non-uniform temperature distributions.

Principal component analysis (PCA) was applied to the time series of the selected temperature sensors 3 and 9 and used to identify UTV and NUTV datasets. The first eigenvalues for the respective datasets were obtained after PCA. The residual eigenvalues were evaluated by subtracting the first eigenvalues from a maximum value of one ($1-E_{val,1N}$). The results of plotting the residual eigenvalues for the respective datasets are depicted in Figure 4. It can be seen that $1-E_{val,1N}$ ranges from 0.0013 to 0.028. The smallest values are associated with measurements on cloudy days, whereas larger values are associated with sunny days. In the latter case, there was partial shading on the measurement device that moved with time during the calibration measurements.

It can be further observed from the figure that there is a jump in the eigenvalues between datasets 10 and 11. This is the boundary where differentiation is made between UTV and NUTV datasets. Based on this, the measurements #1 to #10 were UTV datasets, whereas measurements #11 to #15 were classified as NUTV datasets (Figure 4). It should be noted that the PCA method applied here was designed to cover more than two temperatures for future outlooks.

### 3.3. Estimation of Calibration Parameter Boundaries

**Figure 5:**
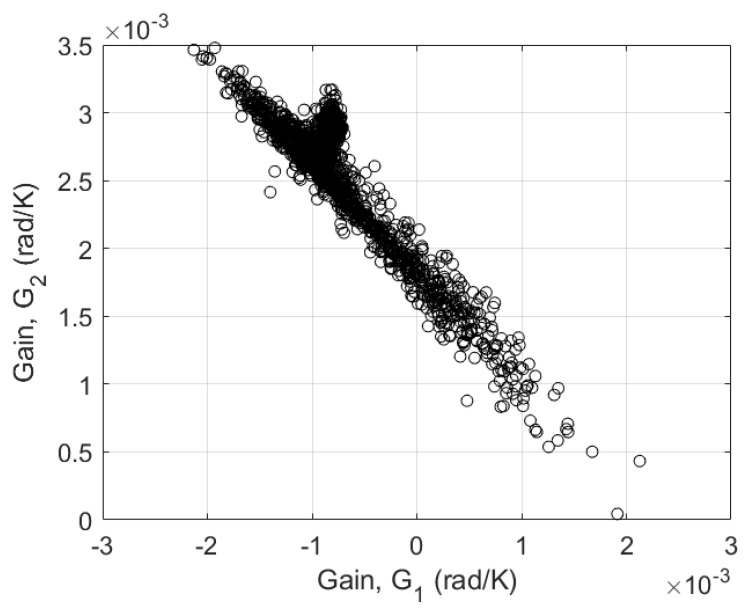
Plot of the correlation between the calibration parameters $G_{1}$ and $G_{2}$ showing all parameter combinations for errors (RMSE) less than 1 mSm^−1^, obtained from fitting dataset #10 with the initial boundaries.

In order to show the strong dependence between individual calibration parameters, an optimization (with calibration strategy type A) was done with the correlation test parameter boundaries shown in Table 1. The results from comparing $G_{1}$ and $G_{2}$ for dataset #10 show a lot of possible solutions where the error is less than 1 mSm^−1^, as shown in Figure 5. For a range of −0.06<G1<0.06 mradK^−1^ and 0<G2<0.1 mradK^−1^, the same minimal fitting errors were obtained. This therefore demonstrates the need to constrain the parameters.

To determine appropriate boundaries for the calibration parameters *G* and τ, the NUTV datasets were used with a reduced temperature range and a linear drift correction model using a broad feasible parameter space (Table 1).After fitting, the minimum and maximum values of $G_{1}$, $G_{2}$, $\tau_{1}$, $\tau_{2}$ were determined and used as the new boundaries of the feasible parameter space for the final calibration (Table 1). It was found that $G_{1}$ is always negative and $G_{2}$ is always positive, with an overall sum of 0.033 mradK^−1^.

### 3.4. Determination of the Representative Calibration Parameters

**Figure 6:**
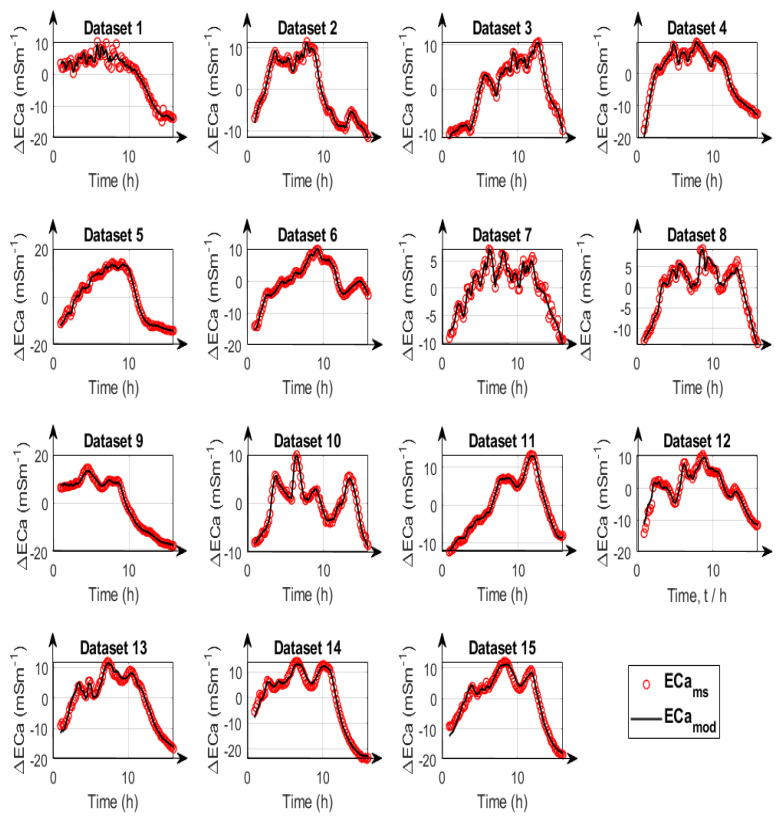
Time series variation of measured apparent electrical conductivity (ECa_ms_) (red circle) and modelled apparent electrical conductivity (ECa_mod_) (black lines) for 15 datasets.

**Figure 7:**
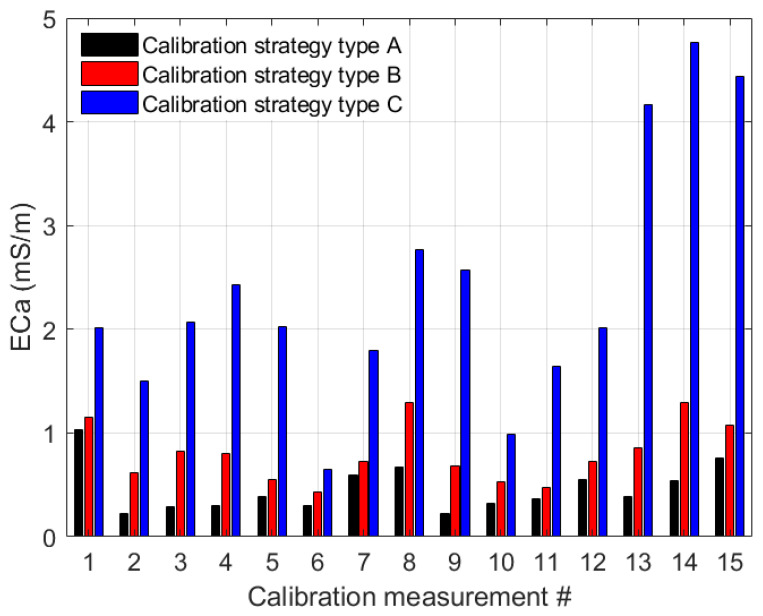
Root mean square errors (RMSE) from fitting with temperature sensors 3 and 9 using calibration strategies A–C (black, red and blue bars, respectively). The black bars show the results of drift correction with calibration parameters obtained from fitting individual measurements. The red bars show the correction with the calibration parameters obtained from simultaneously fitting all datasets. The blue bars are the correction results with parameters obtained from simultaneous fitting with 1 LPF and the mean of temperature sensors 3 and 9.

The reduced feasible parameter space was used to compare the calibration results for calibration strategies A, B, and C. The time series variation of measured and modeled ECa for calibration strategy type A as well as the corrected ECa values estimated for the three calibration strategies are depicted in Figure 6 and Figure 7. The first ten datasets in Figure 7 are UTV datasets, and the remaining five are NUTV datasets.

The results for calibration strategy type A where all parameters were calibrated individually for each dataset show that this strategy provides the best calibration results. The resulting mean RMSE over all datasets is 0.46 mSm^−1^. However, the resulting fitted parameters may not be representative of the entire system drifts because each dataset typically covers a limited temperature range. There is thus a risk that this calibration strategy results in overfitting of the data by accounting for specific pecularities in each dataset. The results for calibration strategy type B which involves simultaneous data fitting with two LPFs showed an overall increase in RMSE values compared with type A. Calibration strategy type B gave a representative parameter set with a mean error of 0.8 mSm^−1^ over all datasets. The results show that type B corrects UTV and NUTV datasets with similar accuracy (Figure 7). The results for calibration strategy type C which involves simultaneous data fitting using only a single LPF gave a mean error of 2.4 mSm^−1^. This shows that the drift correction with one LPF provides a lower accuracy in comparison with two. This is particularly visible in the last 3 NUTV datasets (#13, #14, and #15) where the RMSE values are larger than 4 mSm^−1^ (Figure 7) when a single LPF is used.

With regard to the UTV datasets, fitting with one LPF offers less accurate results than expected, with a mean error of 1.8 mSm^−1^. This is also less accurate in comparison to the results in [24], where the median error is 0.49 mSm^−1^. This is because the $1-E_{val,1N}$ values from the work of [24] are mostly less than 0.0028 across all measurements, which is extremely small in relation to the more complicated datasets in this work.

The calibration parameters obtained from type B and type C are shown in Table 2. It can be seen from the table that when the optimization is done with calibration strategy type B involving two LPFs, two different gains are obtained, one being negative and the other positive. This implies that the system gains ($G_{1}$ and $G_{2}$) partly compensate each other, but only if the times constants ($\tau_{1}$ and $\tau_{2}$) are equal. However, the table shows different time constants, where $LPF_{1}$ has no delay with a corresponding time constant $\tau_{1}$ of 0.002 s and $LPF_{2}$ has a time constant $\tau_{2}$ of 1033 s. Furthermore, it can be seen that $LPF_{1}$ has a strong non-linearity NL with a value of 0.29, whereas the second LPF is linear with a value of 1.02. This shows that it is important to consider these different gains and time constants when fast temperature changes or non-uniform temperature changes occur. Also, the system’s non-linearities must be considered.

For calibration strategy type C, the gain is around the sum of the $G_{1}$ and $G_{2}$ of calibration type B, and NL is 1.48, and the time constant for type C is greater than those in type B. The differences in parameter values between type B and C are likely explained by the fact that the datasets with large non-uniform temperature distributions cannot be properly fitted by calibration type C.

The corresponding gains as ECa values for type B were G1=−0.804 mSm^−1^K^−1^, G2=2.159 mSm^−1^K^−1^ and for type C was G1=1.7 mSm^−1^K^−1^.

Other approaches for temperature drift mitigation rely on the typical static correction methods (without a LPF), where only look-up tables are used to establish unique relationships between temperature and phase. In comparison to these methods, the results in Table 2 show a higher fitting accuracy when LPF is considered, as also confirmed by Tazifor et al. [24].

**Figure 8:**
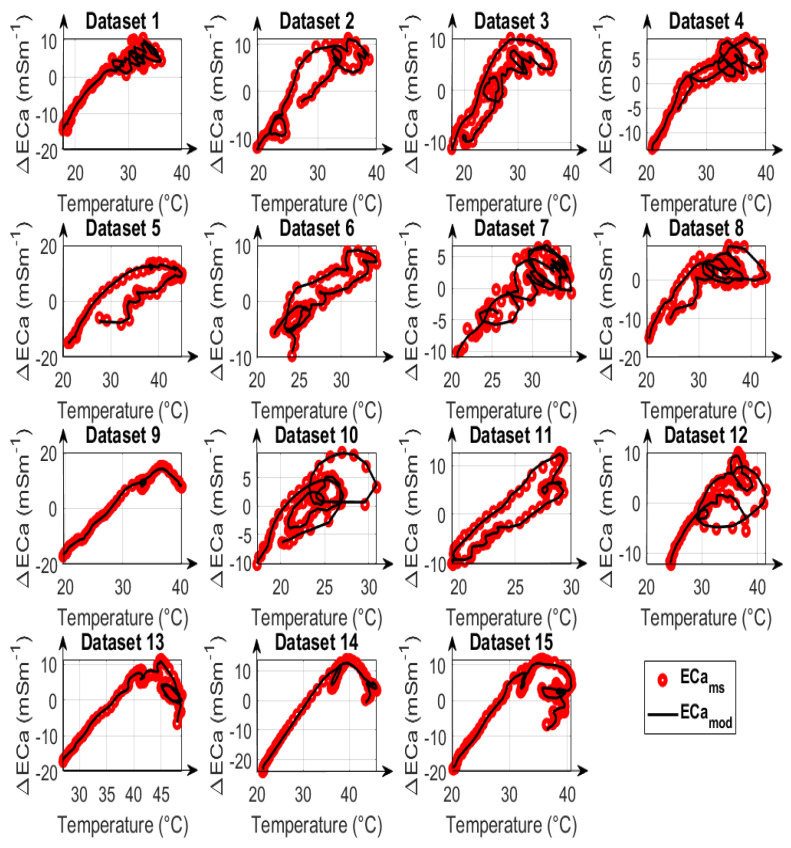
Comparison of modelled apparent electrical conductivity (ECa_mod_), denoted as black lines, with measured apparent electrical conductivity (ECa_ms_), denoted as red circles, as a function of the mean of temperatures 3 and 9 for 15 datasets, using calibration parameters obtained from fitting type A. All ECa values are mean-centered and represented as ECa changes.

**Figure 9:**
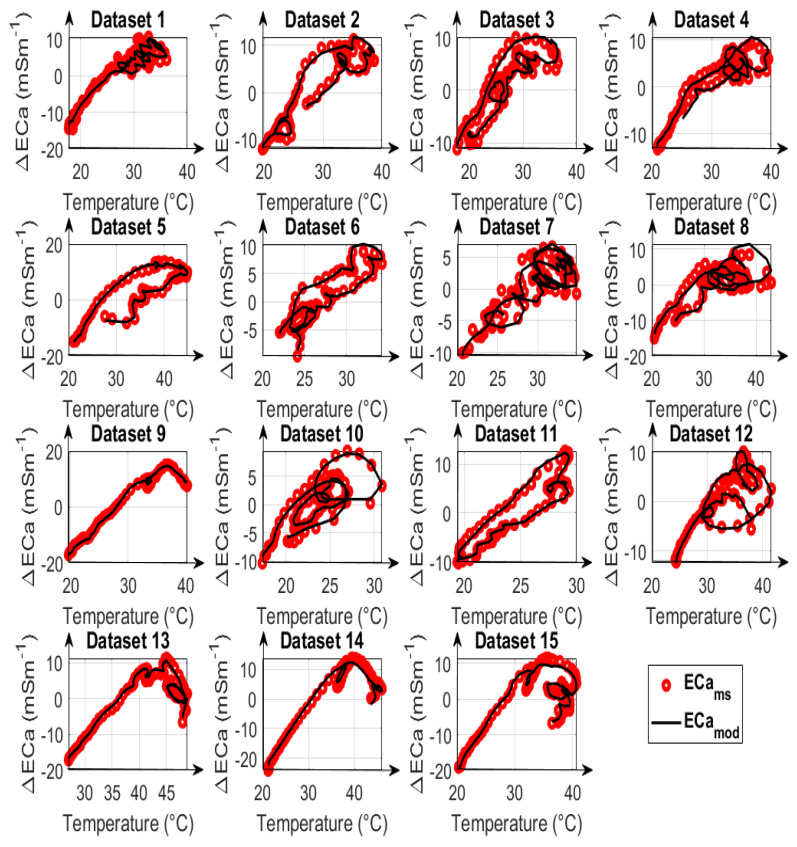
Comparison of modelled apparent electrical conductivity (ECa_mod_), denoted as black lines, with measured apparent electrical conductivity (ECa_ms_), denoted as red circles, as a function of the mean of temperatures 3 and 9 for 15 datasets, using calibration parameters obtained from fitting type B. All ECa values are mean-centered and represented as ECa changes.

**Figure 10:**
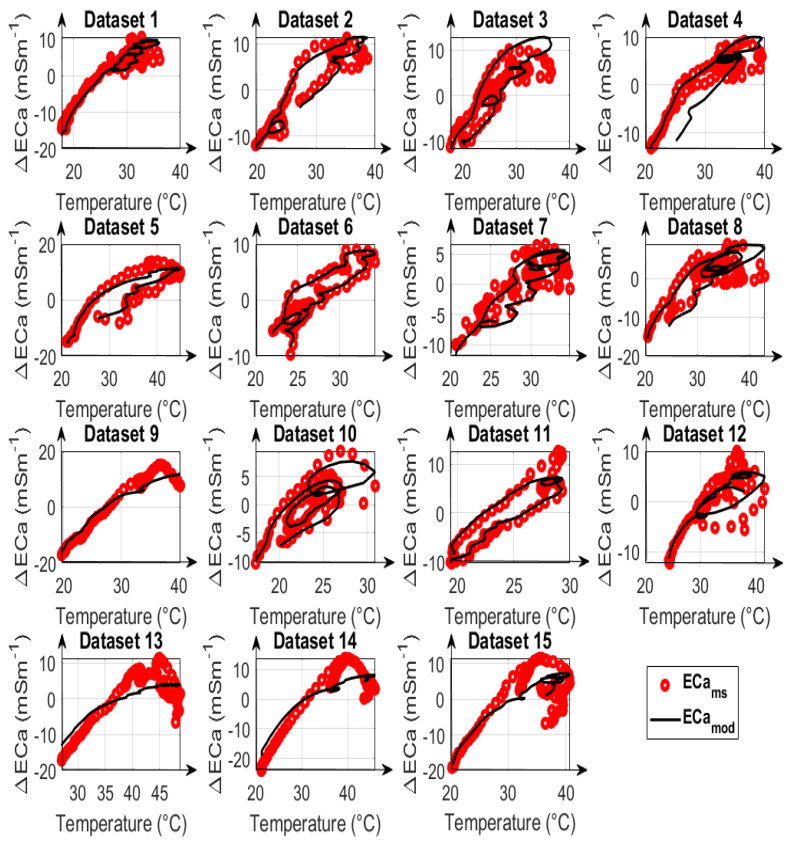
Comparison of modelled apparent electrical conductivity (ECa_mod_), denoted as black lines, with measured apparent electrical conductivity (ECa_ms_), denoted as red circles, as a function of the mean of temperatures 3 and 9 for 15 datasets, using calibration parameters obtained from fitting type C. All ECa values are mean-centered and represented as ECa changes.

In addition to the total error after data correction, the individual measured and modeled data are compared in Figure 8, Figure 9 and Figure 10 for calibration strategies type A, type B, and type C, respectively. It is evident that calibration strategy type A and type B results in accurate fits of the drift model to the measured ECa for datasets with both uniform and non-uniform spatial temperature variations. The hysteresis loops in the relationship between measured ECa values and temperature, which were also reported by Huang et al. [19] and Tazifor et al. [24], are a result of the dynamic heating and cooling history of the system components. They were accurately reproduced by the drift model.

The results obtained from calibration strategy type C with only one LPF are shown in Figure 10. By comparing the ECa_mod_ values with the ECa_ms_ values, it can be seen that the hysteresis effects were best modeled for dataset #6, and that the results are worst for datasets #13, #14, and #15, which are the most complicated NUTV datasets with strong partial shading effects. It is seen clearly here that a drift model with only one LPF can only fit some of the measured data. Overall, the results show that it is possible to correct drift effects resulting from the occurrence of non-uniform temperature variations in measurement systems during EMI data acquisition when two LPFs and two drift-sensitive temperatures are used.

## 4. Conclusions and Discussions

A dynamic drift correction method was presented that uses two low-pass filters (LPF) to model the transient response of electromagnetic induction (EMI) instruments to non-uniform temperature variations. The parameters that control the model are the time constant (τ) from the LPF, the gain (*G*), and the non-linear variable (NL) of the LuT, as well as the system phase offset ($\Phi_{offset}$). In this study, an EMI instrument was used to perform 15 measurements on different days and at different locations. Temperature sensors spread across the device simultaneously measured the ambient internal temperature, varying between 10 °C and 50 °C. To develop a drift correction method, it is necessary to place the temperature sensors in the best positions where sources of drift are expected. The problem here is that the system components have different thermal delays to external temperature change, which leads to the question of whether localized temperature sensors are required to correct the drifts that arise. This study showed that the fastest-reacting sensors can nicely model the thermal delays of the system components with slower reaction times. It is therefore sufficient to place the sensors in the air or on other fast-reacting components like the PCB, where we assume the drifting electronic components to be. For the EMI system used here, there are two drift-sensitive regions, notably, the transmitter region and the receiver region, for an inter-coil spacing of 1.2 m. For these two regions, the temperature sensors 3 and 9 with a quick response were selected.

For a drift model with two or more LPFs, it is difficult to determine calibration parameters through fitting because they are strongly correlated with each other. This creates, on the one hand, the need for an optimization method that searches for the global minimum. To address this, the shuffled complex evolution (SCE-UA) method was used to estimate optimal calibration parameters. On the other hand, the parameter boundaries must be selected carefully since narrow boundaries may lead to a sub-optimum solution and too wide boundaries may lead to convergence problems and a very large computation time. To address this, an initial optimization run was performed by individually fitting each dataset in a linear region. Based on these initial runs, relatively narrow boundaries were derived.

Using these constrained boundaries, the correction with parameters from simultaneously fitting all datasets offered satisfactory results with a mean RMSE of 0.8 mSm^−1^ across all datasets, showing that the parameters obtained are characteristic for the system drifts and that the system can be temperature-calibrated. The final calibrated parameters were G1=−0.804 mSm^−1^K^−1^, G2=2.159 mSm^−1^K^−1^, and $\tau_{1}=0$ s and $\tau_{2}=1030$ s, $NL_{1}=0.326$, and $NL_{2}=1.028$. Here, it should be noted that both positive and negative gains were obtained, which is particularly problematic for drift correction. For slow uniform temperature changes, the gains compensate for each other. However, for fast temperature changes and different time constants, or for non-uniform temperature changes, the presence of both positive and negative gains results in large drift errors if the two different gains are not considered. This implies that it is very important to estimate these gains. The strong non-linearity of $NL_{1}$ shows that a linear model is not sufficient for drift correction, which leads to an increase in dependency between the calibration parameters, so that for each LPF, the NL parameter must also be considered and fitted.

The correction with calibration parameters obtained from using only one LPF while simultaneously fitting all datasets showed that the drift correction was generally less accurate than in the case where two LPFs were used. Due to the non-uniformity of the temperature distribution in the device, the drift model needs more than one temperature sensor for correction. It could be shown for data with extreme non-uniform temperature variations that the ECa error after drift correction with one LPF was very large at about 4.5 mSm^−1^. This situation typically arises when partial shading is experienced during measurements. In order to recognize these situations, it is useful to evaluate the uniformity of temperature variation with principal component analysis (PCA). It is also possible that at least two dominant temperature components are present in the system with different time constants τ and non-linearities NL, which cannot be modelled with only one LPF.

In summary, the dynamic drift correction model with two LPFs provides a reliable solution for removing the effects of temperature-related drifts in a wide range of applications involving near-surface EMI systems. This dynamic correction approach can be subsequently extended to commercial devices by integrating the required temperature sensors, since air temperature sensors are sufficient for the proposed correction method. These sensors can be easily integrated through holes on the devices’ surface. In view of our modular and scalable EMI system under development, only air temperature sensors will be considered for development.

A simple method to calibrate the EMI devices is to perform outdoor measurements. The drift of individual electrical components can be measured in temperature chambers by manufacturers. However, this does not hold true for the coils or for the entire system since such measurements require a metal-free and low-noise environment. Typically, laboratories are not adequate for this. Contrary to the measurement of the drift of single components, the proposed approach is intended to consider the device as an integral drifting system. By incorporating temperature sensors into the instruments and using the new drift correction technique, it is possible to enhance the precision of temperature-related drift correction in EMI systems beyond the level achievable with traditionally used correction techniques. The new method has potential applications in various agricultural scenarios where accurate near-surface ECa measurements are required.

## Figures and Tables

**Table 1:** Boundary for the time constant (τ) and gain (G) calibration parameters before and after fitting.

Parameters	Boundaries	G_1_ (mradK ^−1^ **)**	G_2_ (mradK ^−1^ **)**	τ_1_ (s)	τ_2_ (s)	NL_1_	NL_2_
Correlation	Lower	−0.1	−0.1	0	0	0	0
	Upper	0.1	0.1	4000	4000	2.5	2.5
Initial	Lower	−0.1	−0.1	0	0	1	1
	Upper	0.1	0.1	4000	4000	1	1
Constrained	Lower	−0.06	0.05	0	500	0	0
	Upper	−0.005	0.1	1000	4500	2.5	2.5

**Table 2:** Calibration parameters obtained with calibration strategy type B and type C.

Calibration Strategy Type	G_1_ (mradK ^−1^ )	G_2_ (mradK ^−1^ )	τ_1_ (s)	τ_2_ (s)	NL_1_	NL_2_
B	−0.022	0.061	0.002	1033	0.291	1.02
C	0.048	-	2057	-	1.48	-

## Data Availability

The authors confirm the availability of the measurement data for the reported findings in this work. Data were acquired through measurements performed at the Central Institute of Engineering, Electronics and Analytics (ZEA-2) and are available upon demand.
